# Report of a Case of Placenta Prævia, and One of Shoulder Presentation

**Published:** 1856-04

**Authors:** S. W. Burney

**Affiliations:** Forsyth, Ga.


### ARTICLE II.

*Report of a Case of Placenta Prœvia, and one of Shoulder Presentation.* By S. W. BURNEY, M. D., of Forsyth, Ga.

CASE 1. *Placenta Prœvia.*—On the morning of the 10th of November, 1855, I was sent for, to visit a negro woman belonging to Mr. P———. Reaching her bedside, I learned from the family, she was supposed to be between seven and eight months advanced in her third pregnancy. She was suddenly seized the night before, without any premonition, with uterine hemorrhage. Her owner bled her from the arm, placed her upon the bed, gave thirty drops tinct. opii, and had cold cloths freely used over the uterus. These remedies giving a check to the discharge, her mistress, after giving strict directions to the chief nurse, (a midwife,) to have her called, if the flooding should, at any time recur during the night, retired to rest. Rising at the dawn of day, Mrs. P——— found that her injunctions had been disregarded. The woman had, during the night, lost an alarming quantity of blood, the result of which, was frequent fainting fits—rapid, weak pulse—cold extremities—great difficulty of breathing, and a restlessness, exceeding anything I had ever before witnessed. Suspecting the actual state of affairs, I at once made an examination, and found my fears realized. The placenta seemed to present itself centrally over the os uteri, which was about the size of a shilling-piece, and perfectly dilatable. Labor pains having set in pretty soon after she was attacked, though, now nearly suspended, and other symptoms taken in the aggregate, forced upon my mind the conviction, that nothing but delivery could save the life of the patient.

Requesting Mr. P——— to afford me a consultation, he dispatched, immediately, a message to Dr. S———. Meantime, I used such remedies as her present symptoms seemed to indicate. To relieve the distressing and dangerous jactitation, 80 drops of laudanum was given at a draught; gave brandy toddy pretty freely; mustard used to extremities, and a jug of hot water applied betwixt the shoulders. I also had at hand, a decoction of ergot, to be used whenever reaction took place.

Waiting an hour to witness the effect of the remedies, and finding her somewhat improved in her general condition, the discharge of blood very much lessened, and the tractile efforts of the uterus increased, I decided to give ergot at once. The spurred rye slightly increased the pains; though, as she was comparatively comfortable, I concluded not to disturb her until Dr. S———, who was now looked for every minute, arrived. Dr. S——— was with me in another hour, and making the second examination, we found matters had wonderfully improved. The os uteri was completely dilated; the placental mass wholly detached, and in the vagina, the feet of the child, which we were now satisfied was dead, presented at the orifice of the womb. Concluding to proceed at once to deliver the child, I first, by hooking it with my finger, extracted the placenta; the feet were then taken hold of, and acted upon at every uterine contraction, until the child was delivered. The main difficulty we had to contend with now, was the collapsed state of the system. The brandy toddy and warm applications were directed to be continued; and as the patient had again become restless, the tinct. opii was repeated. Fearing the womb might not contract sufficiently to prevent a return of the hemorrhage, another dose of ergot was administered. This plan succeeded during the evening, in producing a pleasant and equitable temperature, when I felt satisfied of the woman's ultimate recovery, and ventured to leave her in the hands of her nurse.

She subsequently complained of a severe headache, which is invariably the sequence of exhausting, sanquinary discharges. In conclusion, permit me to say, that a somewhat extensive practice of twenty-five years has brought me in contact with only three cases of placenta prœvia; and I sincerely hope it may never be my future, good or bad, to encounter another case. There is no disease to which the female is liable, that is more dangerous; rendered so, many times, by the ignorance and timidity of the attending physician. There is a time, in every case, when the intervention of the practitioner may, by the delivery of the child, save the life of the mother; but let the moment for action pass away unemployed, and death certainly closes the scene. Then, whenever the discharge of blood has been copious enough to make a sufficient impression upon the os tencia, let the physician proceed to deliver instanter. But no man should undertake this process without having, if possible, the concurrence of a brother practitioner of respectable standing. The responsibility attending these cases, is very great; and broad must be the shoulders of that man, who is willing to encounter it, unaided and unassisted. Apply the touch-stone of merit to such as thus court responsibility, and the verdict would be like the handwriting upon the wall. "*Mene, Mene, Tekel Uphursun.*"

CASE 2. *Shoulder Presentation.*—On the morning of the 25th of November last, I was sent for, in great haste, to see a negro woman, belonging to Mr. G———. The distance being only two miles from my residence, I was soon at her bedside. I was informed by her mistress, that the woman was between seven and eight months gone with child; and that she was, from some cause or other, attacked in the night with labor pains. Soon after, she began to flood, which had gone on almost uninterruptedly. Coldness of the extremities had supervened; pulse rapid and weak, with great restlessness, and difficulty of breathing. Upon examination, I found the os dilated to the size of a silver dollar, though somewhat rigid. tinct. opii, mustard, and other appropriate remedies, were instantly used; and plainly perceiving that labor had fairly set in, I determined to rupture the membranes, hoping thereby, to arrest the flooding. The water having escaped the head of the child, as I supposed then, and yet believe, was the presenting part. The only effect, however, was to slightly increase the tractile efforts of the uterus, which had been for several previous hours greatly diminished by the great loss of blood. I now determined to aid Nature, by using ergot in decoction. This article was freely used for an hour or more, with little or no benefit. As the hemorrhage seemed to be upon the increase, without any augmentation of uterine force, I now concluded to make another examination, with the view of determining the then state of affairs. To my consternation and astonishment, I found the right hand and arm of the child, in the vagina. The os tincœ was, however, perfectly relaxed; the index finger of my right hand, was placed between two fingers of the child, with the view of pushing the presenting part back within the cavity of the uterus, which was eventually accomplished. The necessity of delivery being now very apparent, I desisted from using the ergot, and persevered in the use of tinct. opii, spts. nitre, mustard, and toddy, as the pulse seemed to demand it, and ventured to use cold cloths over the region of the womb—moreover, I now had my friend, Dr. S., of Forsyth, sent for to share responsibility, and to assist me in the delivery of the child. The use of the remedies above-mentioned, lessened, to some extent, the flooding, and brought about a partial reaction; inducing me to believe I might await the arrival of Dr. S.

In due season the Doctor came to my aid—at my request he made an examination. He found the arm and hand had again descended, the shoulder presenting at the os uteri. Arrangements were made to deliver forthwith. The woman being in position, oiling well my left hand and arm, I made an effort to reach the feet, but the tonic contraction of the womb, from the long escape of the waters, as well as the effect of ergot, rendered this, in the present state of affairs, wholly impracticable. Another large dose of laudanum was now given; and believing the inhalation of chloroform would greatly assist in relaxing the parts, Dr. S. poured the requisite quantity upon a handkerchief and held it to her nose, until the specific effect was produced. I now made another effort to reach the feet of the child, and although the difficulty had not been entirely overcome, I succeeded, by a persistence in the necessary force, in reaching one of the feet, which was brought down and secured with a fillet. A reasonable amount of labor enabled me to find the other foot, which was also brought down. My hand having lost all feeling, on account of the pressure of the womb, I was now compelled to give way to Dr. S., who, in due time, completed the delivery. It was apprehended, as well by Dr. S., as myself, that the woman would have a slow getting up, in consequence of the great force required to turn the child; but with the assistance of such means as her condition seemed to require, she did very well.

To conclude, I must be permitted to say, I have great confidence in chloroform, as a remedy to overcome excessive uterine contraction. It should, however, on account of its dangerous tendency, be used only in extreme cases; and then, by no one but an experienced physician. The plan of giving this agent in every case of labor, where nothing is to be accomplished or desired, but the diminution of pain, should be reprobated on all hands. I am aware that many scientific and experienced physicians, give it merely to lessen the pain, experienced by most women in labor; gravely assuring us there is no danger in thus using it. But on the other hand, when we remember the many cases of death reported weekly, through the columns of the press, resulting from the administration of this article as an anesthetic, the voice of reason and experience speaks trumpet-tongued against the plan.

